# Efficacy of Repetitive Transcranial Magnetic Stimulation for Acute Central Post-stroke Pain: A Case Study

**DOI:** 10.3389/fneur.2021.742567

**Published:** 2021-11-11

**Authors:** Calogero Malfitano, Angela Rossetti, Stefano Scarano, Chiara Malloggi, Luigi Tesio

**Affiliations:** ^1^Department of Neurorehabilitation Sciences, IRCCS Istituto Auxologico Italiano, Ospedale San Luca, Milano, Italy; ^2^Department of Biomedical Sciences for Health, Università Degli Studi di Milano, Milano, Italy

**Keywords:** thalamic stroke, central post-stroke pain, rTMS, neuropathic pain, cortical excitability, rehabilitation

## Abstract

Although rare, central post-stroke pain remains one of the most refractory forms of neuropathic pain. Repetitive transcranial magnetic stimulation (rTMS) has been reported to be effective in chronic cases. However, there are no data on the effects in the acute and subacute phases after stroke. In this study, we present a case of a patient with thalamic stroke with acute onset of pain and paresthesia who was responsive to rTMS. After a right thalamic stroke, a 32-year-old woman presented with drug-resistant pain and paresthesia on the left side of the body. There were no motor or sensory deficits, except for blunted thermal sensation and allodynia on light touch. Ten daily sessions were performed, where 10 Hz rTMS was applied to the hand area of the right primary motor cortex, 40 days after stroke. Before rTMS treatment (T0), immediately after treatment conclusion (T1), and 1 month after treatment (T2), three pain questionnaires were administered, and cortical responses to single and paired-pulse TMS were assessed. Eight healthy participants served as controls. At T0, when the patient was experiencing the worst pain, the excitability of the ipsilesional motor cortex was reduced. At T1 and T2, the pain scores and paresthesia' spread decreased. The clinical improvement was paralleled by the recovery in motor cortex excitability of the affected hemisphere, in terms of both intra- and inter-hemispheric connections. In this subacute central post-stroke pain case, rTMS treatment was associated with decreased pain and motor cortex excitability changes.

## Introduction

Central post-stroke pain (CPSP) may occur after lesions at any level of the somatosensory pathway of the central nervous system. An incidence of 5–8% has been reported in the first few months after hemispheric stroke (1). Prevalence seems higher in the subacute (from day 15 to day 90) and chronic stages (>90 days after the event) than in the acute phase (2). CPSP is often refractory to standard medical treatments (3). Evidence indicates that repetitive transcranial magnetic stimulation (rTMS) may provide moderate pain relief in intractable chronic CPSP when applied at high frequencies (>5 Hz) over the primary motor cortex (M1) contralaterally to the pain side (4). However, to the best of the authors' knowledge, there are no studies concerning the analgesic effect of rTMS treatment administered in the early subacute phase after stroke. Reasons may include the following: (a) the condition itself is rare; (b) the diagnosis can be difficult when the patient is cognitively impaired and/or; (c) other potential causes of pain may coexist; (d) within a life-threatening condition, pain may not seem a priority to both patients and physicians; (e) first-line therapy for central pain is pharmacological, and rTMS is only considered when drugs are ineffective; (f) there may be contraindications to rTMS, such as a history of epilepsy. This was a case study where clinical changes were correlated with changes in cortical excitability indices.

## Methods

### Participant

**Figure 1:**
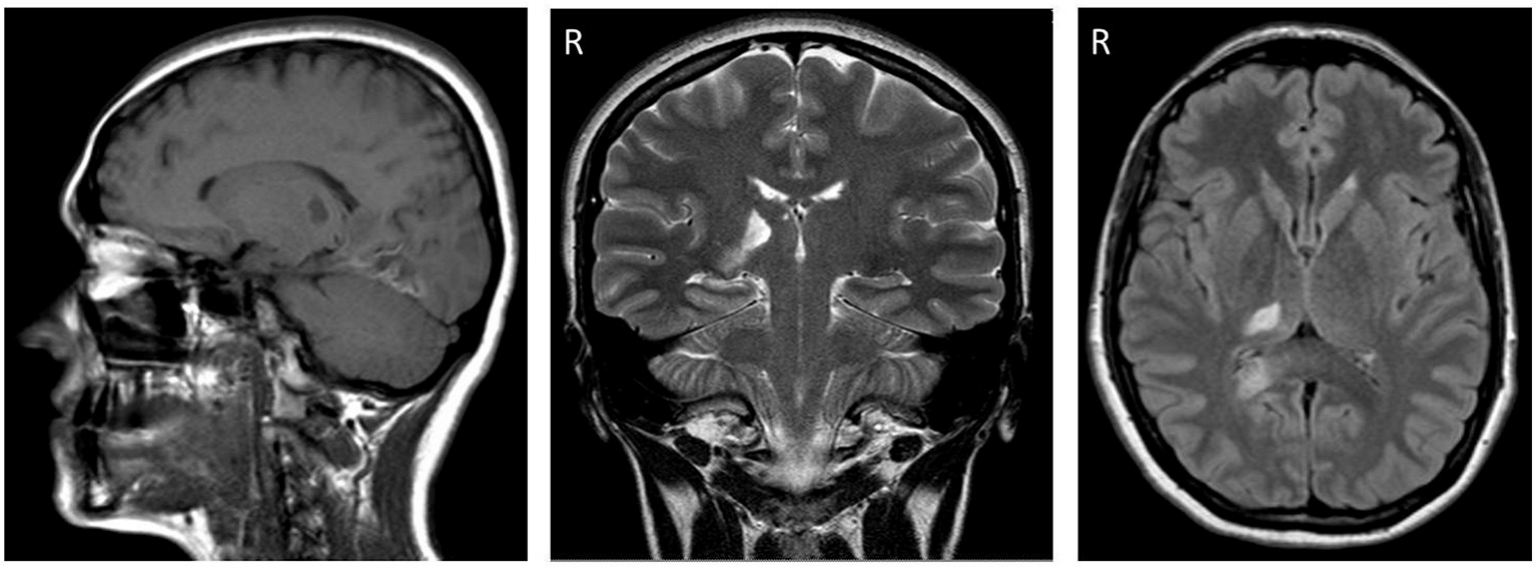
From left to right, sagittal (FLAIR), coronal (T1-weighted), and axial (T1-weighted) sections of brain magnetic resonance imaging (MRI) performed three weeks post-stroke. Note the ischemic lesions affecting the posteroinferior part of the right (R) thalamus and the right occipital lobe.

A 32-year-old woman was admitted to a stroke unit due to acute onset of pain and paresthesia on the left side of her body. Symptoms were associated with mild left hemiparesis and lateral homonymous hemianopia. Her medical history was notable for allergic asthma. The patient was taking a contraceptive pill. The stroke was diagnosed as cryptogenic, although an embolism through a patent foramen ovale was suspected. Brain magnetic resonance imaging (MRI) confirmed an ischemic lesion in the right posteroinferior thalamus, parahippocampal, and occipital cortex (Figure 1).

Twenty-two days after her stroke, the patient was admitted to a Neurorehabilitation unit of a teaching hospital due to persistent pain and paresthesia affecting the left side of her body.

### Clinical Assessment

On admission to the rehabilitation unit, the patient was visited by an experienced physiatrist and diagnosed with CPSP according to the established criteria: (a) development of pain soon after hemispheric ischemic stroke, (b) spontaneous pain and tingling paresthesia with localization consistent with the brain lesion, and (c) exclusion of other causes of pain (5). Extensive cognitive assessment yielded normal results. Neuro-ophthalmologic assessments confirmed left homonymous lateral hemianopia. This symptom was consistent with both thalamic and occipital lobe lesions. No motor deficits or changes in reflexes were observed. Light touch, vibration (tuning fork, 128 Hz) (6), and spatial discrimination of fingers and toes (JVP Dome) (7) were normal. The pain was described as squeezing and pressing. The painful areas showed allodynia upon light touch. The thermal sensation deficit was qualitatively assessed by applying two rollers at 40 and 20°C. All assessments were performed bilaterally. The abnormal sensitivity was determined by comparing the affected and unaffected sides. Diminished sensitivity to cold and heat sensations was revealed on the left side of the body. The patient was right-handed according to the Edinburgh Handedness Questionnaire (8).

Once the patient entered the study, she underwent three more visits: (T0) immediately before the first rTMS treatment, which was performed 40 days after the stroke; (T1) immediately after the last rTMS treatment; (T2) 1 month later (follow-up). In addition, she received pregabalin (300 mg/day) and amitriptyline (20 mg/day) from the first week after stroke throughout the study period.

During each of the three subsequent visits (T0, T1, T2), the patient was asked to rate the mean intensity of pain during the last 24 h on an 11-point (0–10) numerical rating scale (NRS) (no pain to the worst imaginable pain). She was also asked to complete two validated cumulative questionnaires: the Neuropathic Pain Symptom Inventory (NPSI) (9, 10) and the McGill Questionnaire-Short Form (MPQ-sf) (11, 12), both validated in Italian.

The NPSI includes 12 items describing different qualities of the pain experienced in the last 24 h. Ten items characterize the various symptoms on a 0–10 rating scale, for example, *does your pain feel like burning? Does your pain feel squeezing?* Two more items capture the duration of pain on a 0–5 rating scale. Thus, the total NPSI score may range from 0 to 110 (the higher the score, the worse the pain experienced). The MPQ-sf consists of 15 items measuring distinct pain characteristics (11 sensory and 4 affective) during the last week. Items representative of the sensory dimensions are, for instance, *throbbing and shooting*. Items representative of the affective dimension are, for example, *tiring/exhaustive, sickening*, etc. The questionnaire is a rating scale with item scores ranging from 1 to 4 (none/mild/moderate/severe). Thus, the total score may range from 15 to 60 (the higher the score, the worse the pain).

**Figure 2:**
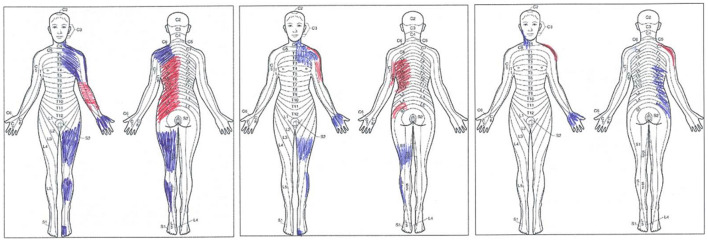
The patient, at three time points, made original drawings. From left to right, drawings collected 40 days after the right thalamic stroke (T0), immediately after the last one of 10 repetitive transcranial magnetic stimulation (rTMS) treatments (T1), and 1 month after the last rTMS session (T2). Pain (red tract) and paresthesia (blue tract) are shown. Note that at T2, the patient acknowledged that she marked the “wrong” side of her body (right rather than left); nevertheless, the original drawing, performed with no cues from the examiner, is provided here.

Finally, the patient was asked to hatch the area of pain on a human outline form after being instructed to differentiate between pain and paresthesia. The patient's drawings are reproduced in Figure 2.

### Assessment of Motor Cortex Excitability

The physician checked for the absence of contraindications to TMS (13). At T0, T1, and T2, the patient performed an extensive electrophysiological test battery. Four indices of intracortical excitability and one of interhemispheric inhibition were computed: (a) resting motor threshold (rMT), (b) short interval intracortical inhibition (SICI), (c) intracortical facilitation (ICF), and (d) ipsilateral silent period (iSP) (two sub-indices computed).

Reference TMS parameters were available from previous studies conducted in the same laboratory. It must be highlighted that throughout this article, the notation of “side” reflects the stimulated hemisphere. Electromyographic potentials were recorded from the hand homolateral to the stimulated hemisphere for iSP tests and from the hand opposite to the stimulated hemisphere for other tests. The complete protocol and related references are presented in the Supplementary Material.

### Stimulation (rTMS)

A physiatrist and neuropsychologist jointly performed the rTMS. An oil-cooled angulated figure-of-eight coil was used (external diameter of each coil 100 mm, model AFEC-02-100-C), connected to a Neuro-MS/D therapeutic variant magnetic stimulator (Neurosoft Ltd., Ivanovo, Russia), which provides repetitive biphasic pulses. The TMS coil was secured using a dedicated coil holder (same as that for motor cortex excitability testing). A published protocol was adopted (14) and performed following the guidelines for safe rTMS use (13). Briefly, 40 trains of 10 Hz rTMS at 90% intensity of rMT were delivered to M1 corresponding to the painful hand (first dorsal interosseous area), each train lasting 5 s, with an intertrain interval of 5 s. Thus, 2,000 pulses were applied in a 6′ 31″ rTMS session. Ten treatments were performed, each on a subsequent working day.

### Study Design and Statistical Approach

An ABB quasi-experimental design was used for this single case (15). Changes in pain scores on NPSI, MPQ-sf, and NRS were considered as independent primary endpoints. Favorable changes in any of these three indices were considered evidence of treatment efficacy.

For pain questionnaires, only individual observations in this single patient were available. Therefore, the approach to statistical significance was based on the logic of comparing individual changes with established thresholds of change for a given test, that is, the “minimal clinically important change” (MCIC). Based on published data, the threshold for “important” change was set to 6 points for the 15–60 MPQ-sf (16); 20% of baseline values for the total score for the NPSI (personal communication, courtesy of Dr. Bouhassira), and 20% (rounded to the nearest integer) of baseline values for the 0–10 NRS (17).

Changes in neurophysiological parameters were considered secondary endpoints. They were considered a form of intra-subject control supporting the non-placebo cause-effect inference on rTMS and pain relief (if any). Given the study design, no statistical analysis could be performed on neurophysiological parameters. Instead, a descriptive data analysis was conducted, including a comparison of baseline data from eight healthy volunteers (mean age 31 ± 4 years; 4 men), providing reference standards for the laboratory. All subjects were right-handed and had no neurological disease.

The study followed the Declaration of Helsinki on human studies (18) and was approved by the institutional ethics committee. The patient provided oral consent for participation in the study, recorded by the principal investigator, and written informed consent to publish the study results. In the Rehabilitation Department, where the study was conducted, rTMS is a treatment that complements many standard motor and cognitive rehabilitation programs. Therefore, the particular disorder treated here (i.e., post-stroke pain) was not deemed to imply contraindications and modalities of application different from those routinely adopted for other impairments, such as post-stroke language impairments (19, 20).

## Results

### Clinical Changes

No adverse events were observed. A clinically important improvement (i.e., a decrement), from NPSI scores of 58 to 41, was detected immediately after the last rTMS session (T1), and maintained until follow-up (T2). Clinically important decrements of the MPQ-sf score (from 42 at T0 to 18 at T2) and NRS scores (from 8 at T0 to 4 at T2) were also recorded. Table 1 provides numeric details. Allodynia on light touch disappeared at T1, while deficits of thermal sensation and the visual field defect remained steady throughout the study period.

**Table 1:** Clinical results at the three time points: 40 days after the right thalamic stroke (T0), immediately after the last repetitive transcranial magnetic stimulation (rTMS) treatment session (T1), and one month after the last rTMS session (T2).

	**T0**	**T1**	**T2**
NPSI (Range 0–110)	58	41^*^	36^∧^
MPQ-sf (Range 15–60)	42	38	18^∧^
NRS (Range 0–10)	8	7	4^∧^

*MPQ-sf, McGill pain questionnaire short-form; NPSI, Neuropathic Pain Symptom Inventory; NRS, Numerical Rating Scale;*

* *Score change exceeding the minimal clinically important change (MCIC) between T0 and T1;*

∧ *Score change exceeding the MCIC between T0 and T2. In all three questionnaires, the lower the score, the better the condition*.

Figure 2 represents the original pain drawings made by the patient. These drawings highlight the progressive reduction of the painful (red tract) and paresthetic (blue tract) areas at the three time points. At T0, pain affected the anterior surface of the forearm and the posterior surface of the trunk on the left side of the body. Tingling paresthesia affected the neck, the shoulder girdle, the lateral side of the arm and the palm, the thigh and the leg of the left lower limb while sparing the knee, and the dorsum of the third, fourth, and fifth toes. Immediately after the last rTMS session (T1), the patient complained of pain affecting the shoulder and the lateral side of the arm and the trunk's posterior surface; overall, paresthesia covered a smaller area. At T2, the pain was confined to the posterolateral surface of the left shoulder only. The former trunk pain had been replaced by tingling paresthesia, which also affected the anterior surface of the neck and the palm.

### Differences in Motor Cortex Excitability Measures

**Figure 3:**
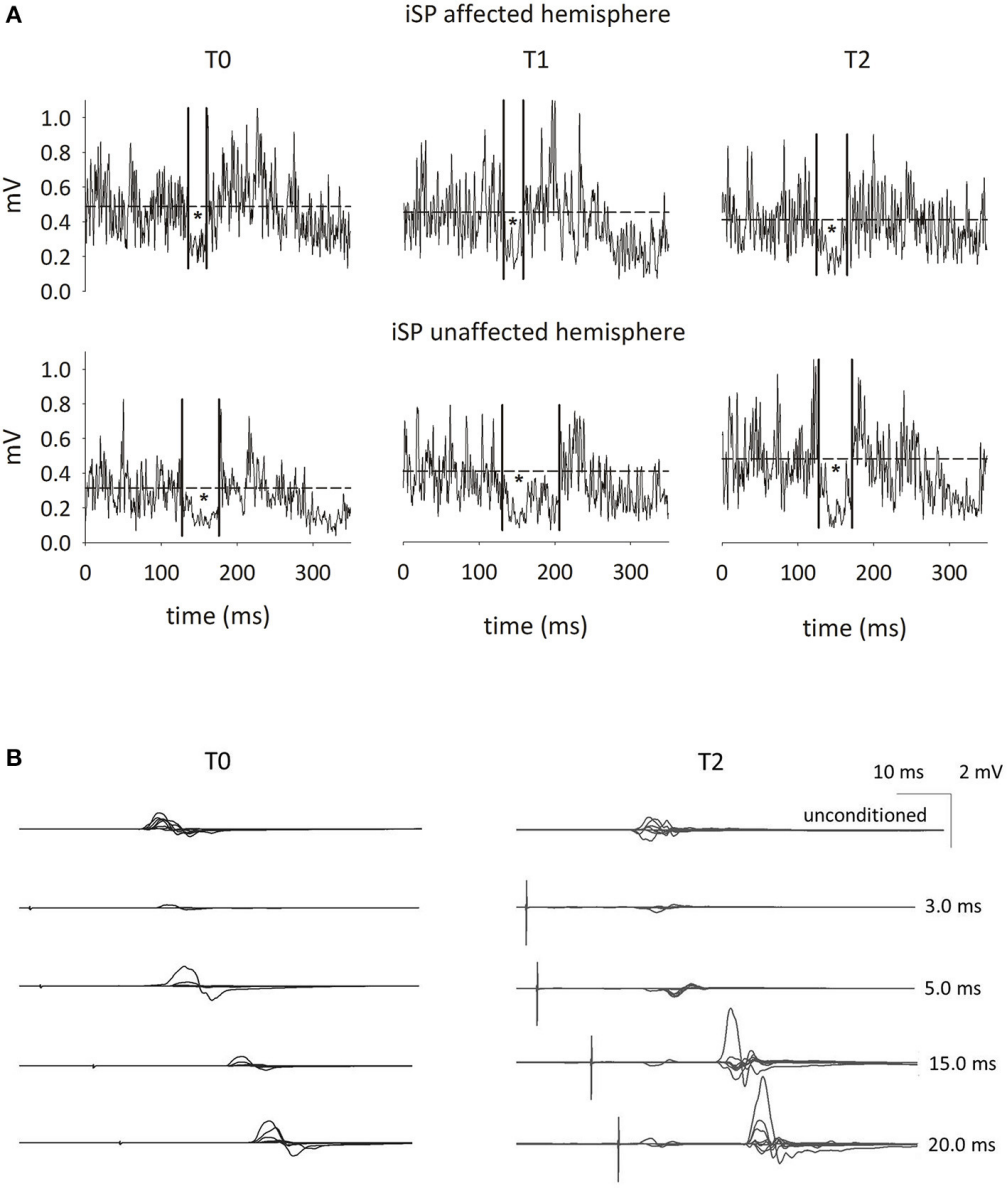
Assessment of motor cortex excitability. **(A)** Shows the ipsilateral silent period (iSP) from TMS suprathreshold stimulation of M1 (120% of resting motor threshold, rMT) of either hemisphere at three time points: 40 days after the right thalamic stroke (T0), immediately after the last rTMS treatment on the primary motor area of the affected hemisphere (T1), and one month after the last rTMS (T2). Surface EMG from the first dorsal interosseous during maximal thumb adduction, rectified, filtered, and averaged across eight stimuli. Dotted lines indicate the mean amplitude of EMG activity in the 10 ms preceding the TMS stimulus. The iSP area is marked with an asterisk (*). See Supplementary Material for further details. **(B)** Shows the short interval intracortical inhibition (SICI) and the intracortical facilitation (ICF) at T0 (left column of tracings) and T2 (right column) of the affected hemisphere. From top to bottom: eight superimposed traces of EMG signals were recorded after a single-pulse TMS delivered at 120% of the resting motor threshold (rMT) (line 1: unconditioned), and after paired-pulse TMS (ppTMS; lines 2–5). For ppTMS, the conditioning stimulus was given at 75% of rMT 3 ms (line 2), 5 ms (line 3), 15 ms (line 4), and 20 ms (line 5) before the conditioned stimulus, delivered at 120% of the rMT.

The numerical results are shown in Supplementary Table 1. Representative tracings are shown in Figure 3. The resting motor threshold did not substantially change at the three time points. The iSP duration was shorter than normal on stimulation of the affected hemisphere and longer on stimulation of the unaffected hemisphere at T0 and T1. At T2, the iSP duration became normal and nearly symmetrical on the stimulation of both sides. At all three time points, the iSP area was greater on stimulation of the unaffected hemisphere when compared to the affected hemisphere, and both hemispheres provided values higher than the control values. SICI was normal bilaterally at all three time points. In contrast, upon stimulation of both hemispheres, ICF was nearly suppressed and even presented as inhibition (see values marked in bold in Supplementary Table 1), both at T0 and T1. ICF regained positive values on stimulation of both hemispheres at T2; however, it showed abnormally high values on stimulation of the unaffected hemisphere.

## Discussion

Despite only including a single case in the present study, this case may extend prior knowledge of rTMS application in patients with CPSP.

A clinically important reduction in CPSP was reported after rTMS treatment administered in the early subacute phase in a patient with an acute onset of pain. This original finding is consistent with previous results in chronic CPSP (21, 22). However, the analgesic effects of rTMS for acute and subacute pain have been less studied. Few studies have been performed on postsurgical pain (23), pain after spinal cord injury (SCI) (24), and experimental model pain. In addition, there are no data available on subacute CPSP (25).

An analgesic effect of rTMS treatment administered to the left dorsolateral prefrontal cortex (DLPFC; one session immediately following surgery and one session 4 h later) was found in patients with pain following gastric bypass in the perioperative setting. Unfortunately, a direct comparison with the present study was prevented due to the difference in the stimulation site (23). In a randomized, double-blind clinical trial, rTMS over the hand area of the M1 alleviated acute neuropathic pain in the early phase of spinal cord injury. However, this effect was limited to 2 or 3 weeks (24). In an experimental model of acute pain (i.e., caused by the application of capsaicin to the skin), high frequency (10 Hz) rTMS applied to the contralateral M1 alleviated the subjective sensation of pain and raise the pain detection thresholds more than sham stimulation. These findings demonstrate the possible analgesic effects of rTMS (25). To date, however, data seem insufficient to prove the effectiveness of rTMS in acute or subacute post-stroke pain. Evidence remains missing regarding the role of laterality, site of stimulation (M1 or DLPFC), and stimulation frequency.

To the best of our knowledge, this is the first study documenting a new pattern of alterations of cortical excitability in response to M1 stimulation in a subacute CPSP patient presenting with neither motor nor sensory deficits except for thermal sensation and abnormalities of light touch sensation. At baseline, the response pattern to paired stimulation at 15 ms and 20 ms interstimulus intervals, usually leading to an increase in the conditioned MEP (ICF) (26), unexpectedly led to a decrease in the MEP. iSP duration was shorter than normal when the affected hemisphere was stimulated, reflecting a lower capacity for interhemispheric inhibition. The iSP was longer than normal on stimulation of the unaffected side.

This response pattern to TMS testing appeared to be the counterpart to pain perception in this patient. Responses can be summarized as a sort of bilateral “pain-related cooling” of intracortical facilitatory circuits, when triggered by stimulation of the motor cortex—which was intact—of the affected hemisphere. This phenomenon is consistent with the fact that (a) ICF and iSP duration, the latter reflecting cortico-cortical excitability, were decreased, whereas rMT, reflecting cortico-spinal excitability, was unaffected; (b) suppression of ICF was bilateral; and (c) the ipsilesional (yet intact) cortex also exerted a low inhibition on the contralesional cortex.

This pattern of response to TMS testing was specific to this patient's lesion. Concerning the bilateral decrease of ICF (even reverting to inhibition), unilateral pain syndrome may lead to bilateral changes in cortical excitability. This was the case for a TMS study of patients with complex regional pain syndrome affecting one hand. These results led to “*the assumption that bilateral cortical disinhibition is not just a secondary phenomenon resulting from pain. It suggests a generalized involvement of the central motor system, which occurs early during the disease and remains present years after its initiation”* (27). However, in that study, SICI depression with no ICF alterations, was recorded.

A possible explanation for these peculiar findings at baseline may be that gamma-aminobutyric acid agonists could exert a bilateral depressive influence selectively on ICF circuits. However, this explanation is unlikely. Such a depressive effect should also be found bilaterally on motor thresholds and iSP duration (28). Furthermore, this patient experienced severe pain, with no sensory deficits involving touch, vibration, or spatial discrimination. Sensory symptoms were confined to the loss of thermal discrimination and allodynia to light touch. Therefore, deafferentation does not appear to be a convincing explanation for pain (28). As a matter of speculation, an alternative hypothesis might be impaired intra-thalamic inhibition.

The mesial (so-called “limbic”) thalamus (29) projects to the anterior cingulate cortex and is considered to control the affective-emotional and behavioral features of pain through this projection. The lateral thalamus is involved in the sensory appraisal of pain through its projections to the parietal cortex, modulating motor cortex excitability (30). The posterior thalamus is also a key station of visual pathways projecting to the visual cortex. The lateral thalamus inhibits the mesial thalamus. For instance, in a single case, a lesion of the lateral thalamus (presumably causing mesial disinhibition) was found to divert sensory stimulation toward crying (31). The lesion observed in the present case (Figure 1), affecting the posteroinferior thalamus, seems consistent with pain/paresthesia syndrome and visual field defects. In short, one can speculate that the damage might have been prevalent for intra-thalamic circuits: these were no longer effective in inhibiting the mesial thalamus, thus leading to severe pain perception and bilateral spreading of the “cooling” of intracortical facilitation through limbic circuits. Most of the lateral thalamus sparing might explain both the scarcity of sensory consequences and the absence of motor deficits.

The effect of rTMS treatment on pain was, in part, expected. From the early '90s, stimulation of M1 through deep brain stimulation, through transcranial magnetic or direct-current stimulation, is known to be effective in treating various pain syndromes (32). Nevertheless, the exact mechanism of action remains unclear. High frequency rTMS on the M1 of the affected side may reinforce the cortical excitability of the affected hemisphere and re-balance inter-hemispheric inhibition (33, 34). Indeed, when the patient was experiencing the worst pain (T0), the excitability of the motor cortex of the affected hemisphere was reduced (i.e., ICF was suppressed and iSP duration was reduced). At T1 and T2, the reduction in pain was paralleled by the recovery in motor cortex excitability of the affected side, as observed by testing intra- and inter-hemispheric connections.

In addition, the pain was relieved mainly in the trunk, despite stimulating the cortical hand area. On the other hand, this finding is consistent with the observation that rTMS was more effective when stimulation was applied over the cortical representation of areas adjacent to, not coincident with, areas of M1 representing the painful body segments (35).

A fundamental limitation of the present results cannot be overlooked, i.e., spontaneous recovery from pain might have occurred, given that stroke was recent. However, this explanation is not entirely satisfactory. CPSP is known to be long-lasting. Contrary to motor and the sensory deficits, it tends to continuously worsen, rather than improve and stabilize in the first months after damage (5). In addition, a prevalent placebo effect is unlikely. Pain and paresthesia improved after rTMS, whereas deficits in thermal sensation and visual field did not show modifications. Further, objective changes in neurophysiologic parameters indeed paralleled the decrease in pain, suggesting that reports of pain perception were credible. Nevertheless, these neurophysiological changes can be the effect rather than the cause of psychological, placebo-induced changes if one accepts a bidirectional brain-mind relationship (36, 37). In the present study, only the iSP was used as a measure of transcallosal inhibition to reduce patient's burden to a minimum. Future studies should also consider alternative interhemispheric communication measures, such as interhemispheric inhibition (IHI) elicited by double TMS coils, testing distinct pathways (38). Another potential limitation of the present study was the use of non-navigated methods for identifying the target area for stimulation, although the impact of neuronavigation on the outcome of rTMS pain therapy has not been established yet (39). Finally, with respect to NPSI pain scores, the MCIC was obtained from personal information by the scale inventor, not from a dedicated psychometric study providing valuable anchors (40). On the other hand, no alternative measures [i.e., the Minimal Detectable Change, or Minimal Real Difference (41)] were available.

In summary, the mechanism behind the effects of the applied rTMS treatment, in this case, remains poorly understood. However, this protocol deserves further investigation for its application in central pain following thalamic stroke.

## Data Availability Statement

The raw data supporting the conclusions of this article will be made available by the authors, without undue reservation.

## Ethics Statement

The studies involving human participants were reviewed and approved by Ethics Committee of Istituto Auxologico Italiano, IRCCS. The patients/participants provided their written informed consent to participate in this study.

## Author Contributions

CMalf and LT contributed to the design of the study. CMalf and AR collected and analyzed the data. CMall processed the data and performed the analysis. CMalf wrote the first draft of the paper. AR and SS revised the draft and wrote the sections of the manuscript. LT revised the paper for intellectual content. All authors contributed to manuscript revision, read, and approved the submitted version.

## Funding

This work was supported by the Istituto Auxologico Italiano, IRCCS, within the RESET research project (Ricerca Corrente 2020, Italian Ministry of Health).

## Conflict of Interest

The authors declare that the research was conducted in the absence of any commercial or financial relationships that could be construed as a potential conflict of interest.

## Publisher's Note

All claims expressed in this article are solely those of the authors and do not necessarily represent those of their affiliated organizations, or those of the publisher, the editors and the reviewers. Any product that may be evaluated in this article, or claim that may be made by its manufacturer, is not guaranteed or endorsed by the publisher.

## References

[1] Andersen G Vestergaard K Ingeman-Nielsen M Jensen TS. Incidence of central post-stroke pain. Pain. (1995) 61:187–93. 10.1016/0304-3959(94)00144-4 7659428

[2] Paolucci S Iosa M Toni D Barbanti P Bovi P Cavallini A. Prevalence and time course of post-stroke pain: a multicenter prospective hospital-based study. Pain Medicine. (2015) 17:924–30. 10.1093/pm/pnv019 26814255

[3] Mulla SM Wang L Khokhar R Izhar Z Agarwal A Couban R. Management of central poststroke pain: systematic review of randomized controlled trials. Stroke. (2015) 46:2853–60. 10.1161/STROKEAHA.115.010259 26359361

[4] Leung A Shirvalkar P Chen R Kuluva J Vaninetti M Bermudes R. Transcranial magnetic stimulation for pain, headache, and comorbid depression: INS-NANS expert consensus panel review and recommendation. Neuromodulation. (2020) 23:267–90. 10.1111/ner.13094 32212288

[5] Klit H Finnerup NB Jensen TS. Central post-stroke pain: clinical characteristics, pathophysiology, and management. Lancet Neurol. (2009) 8:857–68. 10.1016/S1474-4422(09)70176-0 19679277

[6] Pestronk A Florence J Levine T Al-Lozi MT Lopate G Miller T. Sensory exam with a quantitative tuning fork: rapid, sensitive and predictive of SNAP amplitude. Neurology. (2004) 62:461–4. 10.1212/01.WNL.0000106939.41855.36 14872031

[7] Van Boven R Johson KO. The limit of tactile spatial resolution in humans: grating orientation discrimination at the lip, tongue, and finger. Neurology. (1995) 44:2361–6. 10.1212/WNL.44.12.2361 7991127

[8] Oldfield RC. The assessment and analysis of handedness: the Edinburgh inventory. Neuropsychologia. (1971) 9:97–113. 10.1016/0028-3932(71)90067-4 5146491

[9] Bouhassira D Attal N Fermanian J Alchaar H Gautron M Masquelier E. Development and validation of the neuropathic pain symptom inventory. Pain. (2004) 108:248–57. 10.1016/j.pain.2003.12.024 15030944

[10] Padua L Briani C Jann S Nobile-Orazio E Pazzaglia C Morini A. Validation of the Italian version of the neuropathic pain symptom inventory in peripheral nervous system diseases. Neurol Sci. (2009) 30:99–106. 10.1007/s10072-009-0025-y 19198756

[11] Grafton K V Foster NE Wright CC. Test-retest reliability of the Short-Form McGill Pain Questionnaire: assessment of intraclass correlation coefficients and limits of agreement in patients with osteoarthritis. The Clinical journal of pain. (2005) 21:73–82. 10.1097/00002508-200501000-00009 15599134

[12] Maiani G Sanavio E. Semantics of pain in Italy: the Italian version of the McGill Pain Questionnaire. Pain. (1985) 22:399–405. 10.1016/0304-3959(85)90045-4 4047708

[13] Rossi S Hallett M Rossini PM Pascual-Leone A Avanzini G Bestmann S. Safety, ethical considerations, and application guidelines for the use of transcranial magnetic stimulation in clinical practice and research. Clin Neurophysiol. (2009) 120:2008–39. 10.1016/j.clinph.2009.08.016 19833552 PMC3260536

[14] Lefaucheur J-P Ayache SS Sorel M Farhat WH Zouari HG Ciampi de Andrade D. Analgesic effects of repetitive transcranial magnetic stimulation of the motor cortex in neuropathic pain: influence of theta burst stimulation priming. Eur J Pain. (2012) 16:1403–13. 10.1002/j.1532-2149.2012.00150.x 22508405

[15] Kazdin AE. Single-Case Research Designs: Methods for Clinical and Applied Settings. New York, NY: Oxford University Press (2011).

[16] Strand LI Ljunggren AE Bogen B Ask T Johnsen TB. The Short-Form McGill Pain Questionnaire as an outcome measure: test-retest reliability and responsiveness to change. Eur J Pain. (2008) 12:917–25. 10.1016/j.ejpain.2007.12.013 18289893

[17] Farrar John T Young James P LaMoreaux Linda Werth John L Poole R Michael. Clinical importance of changes in chronic pain intensity measured on an 11-point numerical pain rating scale. Pain. (2001) 94:149–58. 10.1016/S0304-3959(01)00349-9 11690728

[18] WM Association. WMA Declaration of Helsinki – ethical principles for medical research involving human subjects. JAMA. (2013) 310:2191–4. 10.1001/jama.2013.281053 24141714

[19] Rossetti A Malfitano C Malloggi C Banco E Rota V Tesio L. Phonemic fluency improved after inhibitory transcranial magnetic stimulation in a case of chronic aphasia. Int J Rehabil Res. (2019) 42:92–5. 10.1097/MRR.0000000000000322 30300167 PMC6382039

[20] Malfitano C Banco E Rossetti A Casati C Malloggi C Scarano S. rTMS can improve post-stroke apraxia of speech. A case study. Brain Stimul. (2019) 12:380–2. 10.1016/j.brs.2018.12.006 30559001

[21] Rasche D Ruppolt M Stippich C Unterberg A Tronnier VM. Motor cortex stimulation for long-term relief of chronic neuropathic pain: a 10 year experience. Pain. (2006) 121:43–52. 10.1016/j.pain.2005.12.006 16480828

[22] Young NA Sharma M Deogaonkar M. Transcranial magnetic stimulation for chronic pain. Neurosurg Clin North Am. (2014) 25:819–32. 10.1016/j.nec.2014.07.007 25240669

[23] Borckardt JJ Reeves ST Kotlowski P Abernathy JH Field LC Dong L. Fast left prefrontal rTMS reduces post-gastric bypass surgery pain: findings from a large-scale, double-blind, sham-controlled clinical trial. Brain Stimul. (2014) 7:42–8. 10.1016/j.brs.2013.07.007 24527503

[24] Zhao CG Sun W Ju F Wang H Sun XL Mou X. Analgesic effects of directed repetitive transcranial magnetic stimulation in acute neuropathic pain after spinal cord injury. Pain Med. (2020) 21:1216–23. 10.1093/pm/pnz290 31722404

[25] Sacco P Prior M Poole H Nurmikko T. Repetitive transcranial magnetic stimulation over primary motor vs non-motor cortical targets; effects on experimental hyperalgesia in healthy subjects. BMC Neurol. (2014) 14:1–8. 10.1186/s12883-014-0166-3 25182028 PMC4163168

[26] Talelli P Greenwood RJ Rothwell JC. Arm function after stroke: neurophysiological correlates and recovery mechanisms assessed by transcranial magnetic stimulation. Clin Neurophysiol. (2006) 117:1641–59. 10.1016/j.clinph.2006.01.016 16595189

[27] Schwenkreis P Janssen F Rommel O Pleger B Völker B Hosbach I. Bilateral motor cortex disinhibition in complex regional pain syndrome (CRPS) type I of the hand. Neurology. (2003) 61:515–9. 10.1212/WNL.61.4.515 12939426

[28] Kaelin-Lang A Luft AR Sawaki L Burstein AH Sohn YH Cohen LG. Modulation of human corticomotor excitability by somatosensory input. J Physiol. (2002) 540:623–33. 10.1113/jphysiol.2001.012801 11956348 PMC2290238

[29] Vertes RP Linley SB Hoover WB. Limbic circuitry of the midline thalamus. Neurosci Biobehav Rev. (2015) 54:89–107. 10.1016/j.neubiorev.2015.01.014 25616182 PMC4976455

[30] Cappe C Morel A Barone P Rouiller EM. The thalamocortical projection systems in primate: an anatomical support for multisensory and sensorimotor interplay. Cerebral Cortex. (2009) 19:2025–37. 10.1093/cercor/bhn228 19150924 PMC2722423

[31] Bassani R Rosazza C Ghirardin L Caldiera V Banco E Casati C. Crying spells triggered by thumb-index rubbing after thalamic stroke: a case report. BMC Res Notes. (2017) 10:109. 10.1186/s13104-017-2425-z 28235422 PMC5326498

[32] Morishita T Inoue T. Brain stimulation therapy for central post-stroke pain from a perspective of interhemispheric neural network remodeling. Front Hum Neurosci. (2016) 10:1–8. 10.3389/fnhum.2016.00166 27148019 PMC4838620

[33] Cunningham DA Machado A Janini D Varnerin N Bonnett C Yue G. Assessment of inter-hemispheric imbalance using imaging and noninvasive brain stimulation in patients with chronic stroke. Arch Phys Med Rehabil. (2015) 96:S94–103. 10.1016/j.apmr.2014.07.419 25194451 PMC4348350

[34] Kubis N. Non-invasive brain stimulation to enhance post-stroke recovery. Front Neural Circ. (2016) 10:56. 10.3389/fncir.2016.00056 27512367 PMC4961863

[35] Lefaucheur JP Drouot X Menard-Lefaucheur I Keravel Y Nguyen JP. Motor cortex rTMS restores defective intracortical inhibition in chronic neuropathic pain. Neurology. (2006) 67:1568–1574. 10.1212/01.wnl.0000242731.10074.3c 17101886

[36] Tesio L Scarano S. Ground walking in chronic complete spinal cord injury: does epidural stimulation allow “awakening” of corticospinal circuits? A wide-ranging epistemic criticism. Am J Phys Med Rehabil. (2021) 100:e43–7. 10.1097/PHM.0000000000001520 32618753 PMC7969152

[37] Tesio L Buzzoni M. The illness-disease dichotomy and the biological-clinical splitting of medicine. Med Hum. (2020) 1–6. 10.1136/medhum-2020-011873 32994200 PMC8639948

[38] Boddington LJ Reynolds JNJ. Targeting interhemispheric inhibition with neuromodulation to enhance stroke rehabilitation. Brain Stimul. (2017) 10:214–22. 10.1016/j.brs.2017.01.006 28117178

[39] Lefaucheur JP Nguyen JP. A practical algorithm for using rTMS to treat patients with chronic pain. Neurophysiol Clin. (2019) 49:301–7. 10.1016/j.neucli.2019.07.014 31375381

[40] Caronni A Picardi M. Letter to the editor concerning the article: “a prospective study to establish the minimal clinically important difference of the Mini-BESTest in individuals with stroke.” Clin Rehabil. (2021) 2:2692155211040733. 10.1177/02692155211040733 34472986

[41] Tesio L. Outcome measurement in behavioural sciences: a view on how to shift attention from means to individuals and why. Int J Rehabil Res. (2012) 35:1–12. 10.1097/MRR.0b013e32834fbe89 22315141

