# Supplementary material for: Efficacy of Repetitive Transcranial Magnetic Stimulation for Acute Central Post-stroke Pain: A Case Study
Source: Front Neurol. 2021 Nov 11;12:742567. doi: 10.3389/fneur.2021.742567 (PMC8631781; doi:10.3389/fneur.2021.742567)
Supplement: Supplementary file 1 [file Table_1.DOCX]

Supplementary Material

# Protocol for the assessment of motor cortex excitability

The excitability of the motor cortex (M1 area) was assessed by single or paired-pulse TMS using a figure-of-eight coil (external diameter of each coil 100 mm, model FEC-02-100). The coil was connected to a monophasic Neuro-MS/D Magnetic Stimulator (Neurosoft Ltd., Ivanovo, Russia; max peak field intensity 2.0 T).

A dedicated coil holder secured the TMS coil. The patient was seated on a comfortable chair and asked to keep her hands relaxed on the thighs. A headrest prevented head movements and displacement of the stimulus site during TMS testing.

Surface electromyography (sEMG) responses, that is, motor evoked potentials (MEPs), were recorded using surface electrodes (15 × 20 mm, Spes Medica S.r.l., Italy) placed on the first dorsal interosseous (FDI) muscle (belly tendon arrangement). EMG signals were amplified, filtered (band-pass 5 Hz–10 kHz), and A-D converted (sampling frequency 25 kHz) by a Neuro MEP Micro EMG device (Neurosoft Ltd., Ivanovo, Russia).

Four excitability indices were computed:

1. The hot spot was found by delivering a series of suprathreshold stimuli as the coil was systematically moved over the scalp until the most prominent MEP was elicited from the FDI. The resting motor threshold (rMT) was determined by reducing the stimulus intensity in steps of 1% of the maximum stimulator output. The rMT was defined as the lowest stimulation intensity evoking an MEP >50 µV at least 5 out of 10 times under complete muscle relaxation. Complete FDI relaxation was monitored using sEMG (1).
2. Short-interval intracortical inhibition (SICI).
3. Intracortical facilitation (ICF). SICI and ICF were measured using paired-pulse stimulation, following Kujirai et al. (2). The conditioning stimulus was set at 75% of the rMT, and the test stimulus was set at 120% of the rMT. The interstimulus intervals (ISI) were set at 3 ms and 5 ms for SICI, and 15 ms and 20 ms for ICF. Eight pairs of pulses were delivered for each ISI in a random sequence. Four stimuli at 120% of rMT at the beginning and four at the end of the sequence were also delivered (MEP_nonconditioned_) (total = 40 stimuli). The SICI and ICF were standardized as follows:

$$\mathrm{SICI} or \mathrm{ICF}=\left| 100*\left[ 1 - \left( \frac{\mathrm{MEP}_{\mathrm{conditioned}}}{\mathrm{MEP}_{\mathrm{nonconditioned}}} \right) \right] \right|$$

4. The ipsilateral Silent Period (iSP) was tested as previously described by Trompetto et al. (3). Briefly, the sEMG from the FDI was recorded (band-pass 10-1000 Hz; sampling rate 2 kHz) during maximal voluntary thumb adduction. Approximately 1 s after effort initiation, a supramaximal stimulus (about 140% of the rMT) was applied to the homolateral cortical hot spot, causing a transient and partial “silencing” of the ipsilateral EMG signal. Silencing is the result of inhibition of the contralateral motor cortex through callosal pathways. Eight EMG signals were recorded, and off-line rectified, filtered, and averaged. Two parameters were then considered representative of the strength of interhemispheric inhibition: the mean (SD) iSP duration and the mean (SD) absolute iSP area. iSP onset was defined as the time point after the TMS stimulus when the EMG activity was lower than the mean amplitude of the EMG activity in the 10 ms preceding the TMS stimulus (EMG_mean_). iSP ending was defined as the first time point after iSP onset, when the EMG activity returned above EMG_mean_. Therefore, iSP duration was defined as (iSP ending – iSP onset). The iSP area was calculated by averaging the differences between EMG_mean_ and EMG activities at each iSP point multiplied by the iSP duration. The longer the iSP duration and the higher the iSP area, the higher the inhibition.

**References:**

1. Rothwell JC, Hallett M, Berardelli A, Eisen A, Rossini P, Paulus W. Magnetic stimulation: motor evoked potentials. The International Federation of Clinical Neurophysiology. *Electroencephalogr Clin Neurophysiol Suppl* (1999) **52**:97–103.

2. Kujirai T, Caramia MD, Rothwell JC, Day BL, Thompson PD, Ferbert A, Wroe S, Asselman P, Marsden CD. Corticocortical inhibition in human motor cortex. *J Physiol* (1993) **471**:501–19. doi:10.1113/jphysiol.1993.sp019912

3. Trompetto C, Bove M, Marinelli L, Avanzino L, Buccolieri A, Abbruzzese G. Suppression of the transcallosal motor output: A transcranial magnetic stimulation study in healthy subjects. *Exp Brain Res* (2004) **158**:133–140. doi:10.1007/s00221-004-1881-6

# Supplementary Table

**Table s1** Neurophysiological parameters

**A1) Affected hemisphere T0**

|  | **SICI3** | **SICI5** | | **SICI3 + 5** | | **ICF15** | | **ICF20** | | **ICF15 + 20** |
| --- | --- | --- | --- | --- | --- | --- | --- | --- | --- | --- |
|
| **Median** | **100.0** | **96.8** | | **100.0** | | **89.4** | | **85.1** | | **86.8** |
| **Q1–Q3** | **100.0**–**100.0** | **90.4**–**100.0** | | **93.3**–**100.0** | | **75.4**–**100.0** | | **73.2**–**96.4** | | **73.2-97.5** |
| **A2) Affected hemisphere T1** | | |  | |  | |  | |
| **Median** | **100.0** | **63.9** | | **92.2** | | **67.7** | | 21.9 | | **57.1** |
| **Q1–Q3** | **92.5***–***100.0** | **39.8***–***89.5** | | **67.6***–***100.0** | | **50.9***–***78.6** | | 6.2–64.4 | | **12.0***–***71.0** |
| **A3) Affected hemisphere T2** | | |  | |  | |  | |
| **Median** | **88.8** | **39.0** | | **79.9** | | 18.5 | | 77.4 | | 27.9 |
| **Q1–Q3** | **70.1***–***100.0** | **17.8***–***83.8** | | **29.8***–***92.4** | | 9.7–28.4 | | 37.5–110.2 | | 19.6–73.6 |
| **A4) Unaffected hemisphere T0** | | |  | |  | |  | |
| **Median** | **100.0** | **95.0** | | **100.0** | | **88.8** | | **82.0** | | **87.5** |
| **Q1–Q3** | **100.0***–***100.0** | **75.5***–***100.0** | | **89.6***–***100.0** | | **58.4***–***96.1** | | **64.3***–***89.5** | | **59.3***–***91.8** |
| **A5) Unaffected hemisphere T1** | | |  | |  | |  | |
| **Median** | **100.0** | **79.2** | | **100.0** | | **76.2** | | **72.9** | | **73.7** |
| **Q1–Q3** | **100.0***–***100.0** | **62.9***–***93.0** | | **80.0***–***100.0** | | **25.5***–***85.8** | | **40.0***–***89.7** | | **32.7***–***88.1** |
| **A6) Unaffected hemisphere T2** | | |  | |  | |  | |
| **Median** | **100.0** | **89.7** | | **100.0** | | 492.1 | | 484.3 | | 484.3 |
| **Q1–Q3** | **88.3***–***100.0** | **63.5***–***109.9** | | **70.1***–***100.0** | | 176.0–785.0 | | 432.2–721.5 | | 294.3–785.0 |

**A7) Controls**

| **Median** | **100.0** | **84.8** | **94.5** | 6.9 | 8.1 | 16.2 |
| --- | --- | --- | --- | --- | --- | --- |
| **Q1–Q3** | **86.9***–***100.0** | **68.0***–***87.7** | **79.8***–***98.7** | **49.5***–*153.7 | **43.2***–*62.0 | **48.9***–*67.5 |

**B1) Patient**

| **Hemisphere** | **Time Point** | **rMT** | **iSP duration (s)** | **iSP Area**  **(mV * s)** |
| --- | --- | --- | --- | --- |
| **Affected** | T0 | 44 | 24.0 | 5.1 |
|  | T1 | 42 | 26.0 | 4.9 |
|  | T2 | 46 | 40.5 | 5.4 |
| **Unaffected** | T0 | 44 | 48.5 | 7.3 |
|  | T1 | 50 | 75.5 | 14.2 |
|  | T2 | 53 | 44.0 | 10.7 |

**B2) Control values**

|  | **rMT** | **iSP duration (s)** | **iSP Area**  **(mV * s)** |
| --- | --- | --- | --- |
| **Median** | 42.5 | 30.8 | 3.3 |
| **Q1–Q3** | 38.3–51.0 | 27.3–38.4 | 2.8–4.0 |

Rows labeled A show results from the intracortical excitability tests: short-interval intracortical inhibition (SICI) and intracortical facilitation (ICF) tests. Tables A1–A2–A3: results from the affected hemisphere. Tables A4–A5–A6: results from the unaffected hemisphere. Table A7: results from the Control Group (n = 8). SICI and ICF values represent the percentage of change induced by the conditioning stimulus on the conditioned stimulus. Data are presented as the median and interquartile range (Q1 and Q3: first and third quartiles of distribution). Values marked in bold represent inhibition. SICI3 and SICI5 represent SICI for interstimulus intervals of 3 ms and 5 ms, respectively. ICF15 and ICF20 represent ICF for interstimulus intervals of 15 ms and 20 ms, respectively. For tests on SICI, values for interstimulus intervals of 3 ms and 5 ms were grouped and median values are presented (SICI 3 + 5); for tests on ICF, values for interstimulus intervals of 15 ms and 20 ms were grouped and median values are presented (ICF 15 + 20).

Rows labeled B show the resting motor threshold (rMT) and the results from the interhemispheric inhibition test: ipsilateral silent period (iSP). For iSP, data on duration and area are provided. Table B1: results from the patient’s affected and unaffected hemispheres. Table B2: results from the control group (n = 8).
